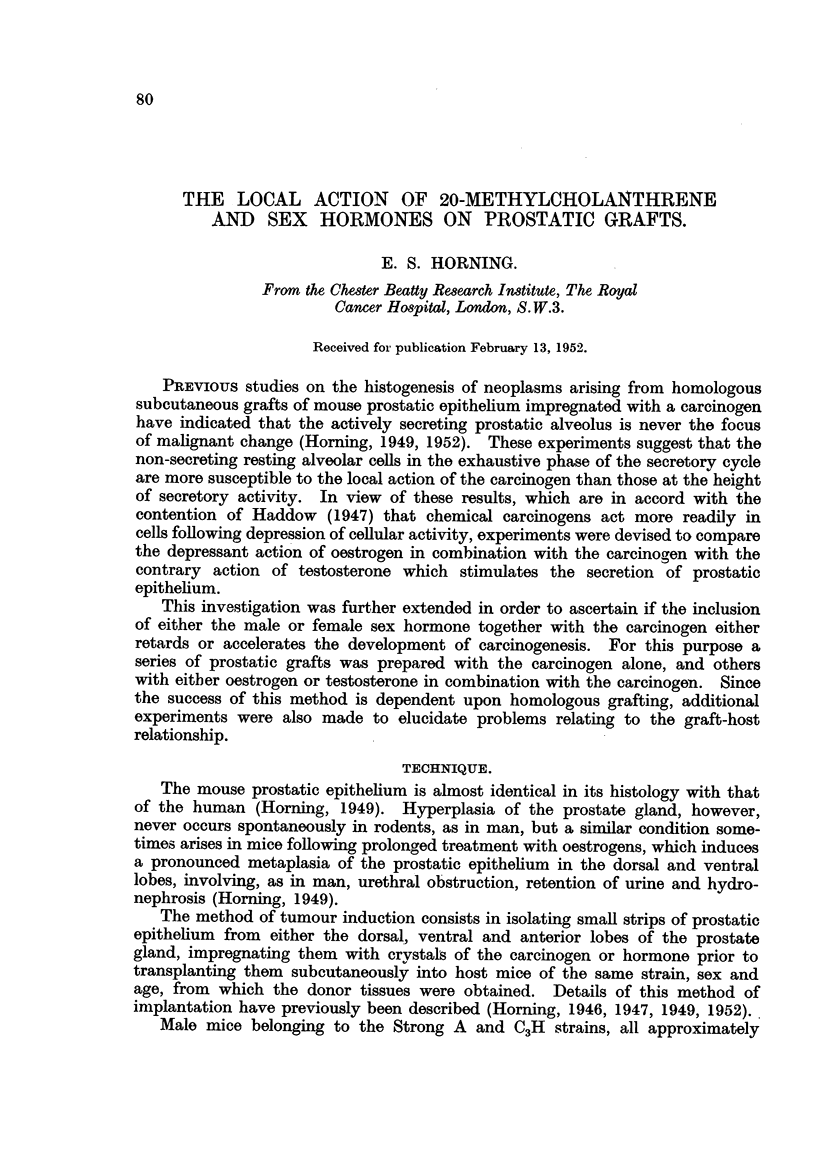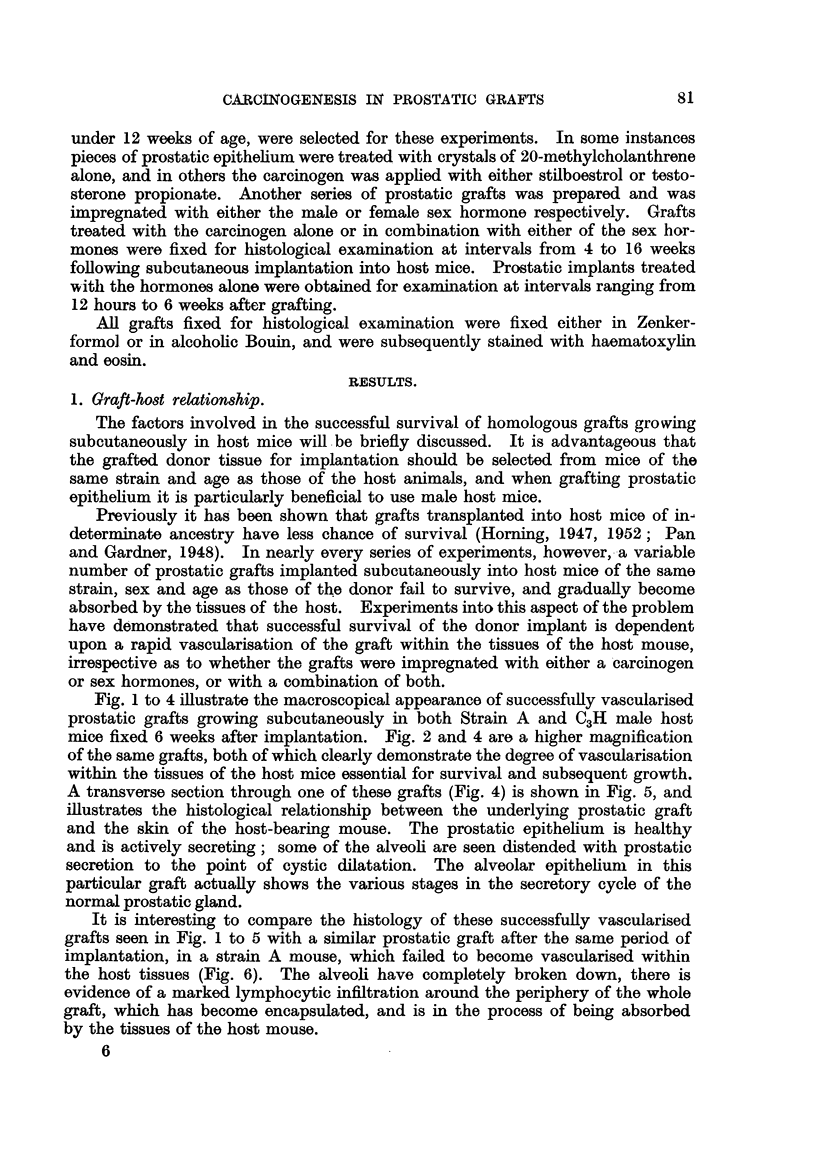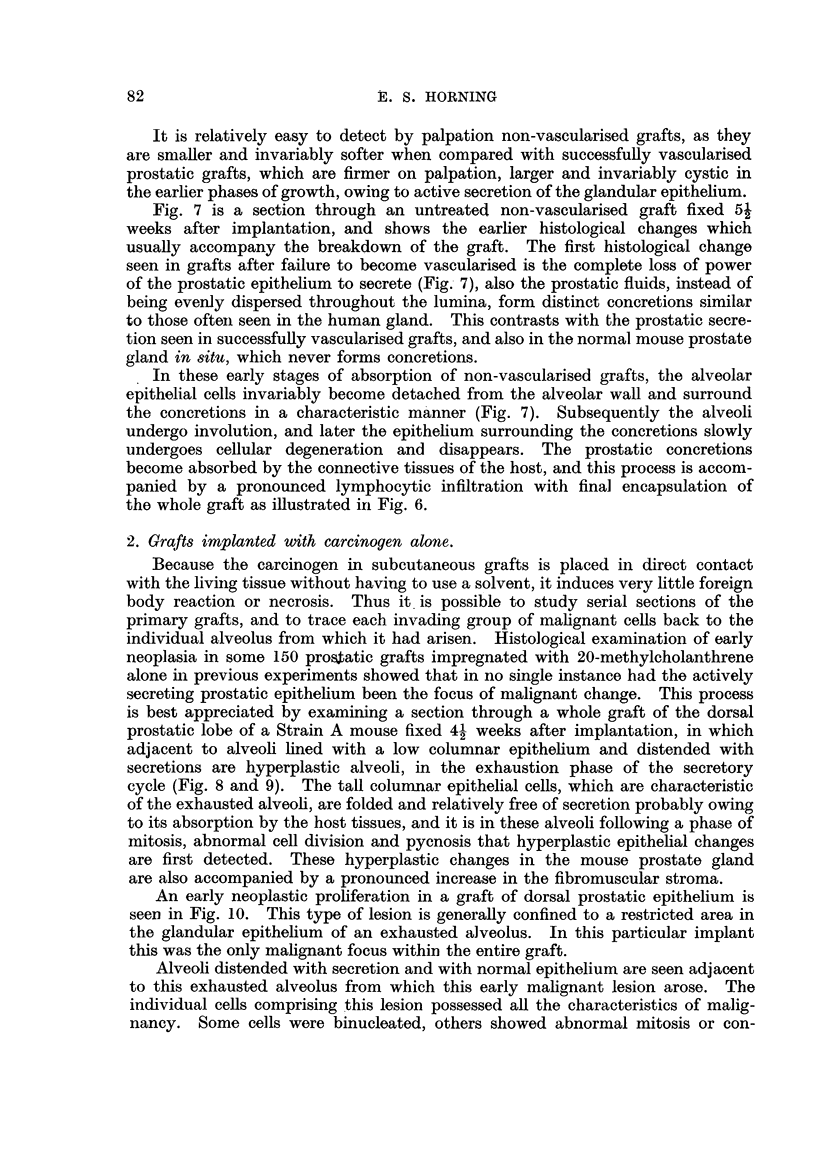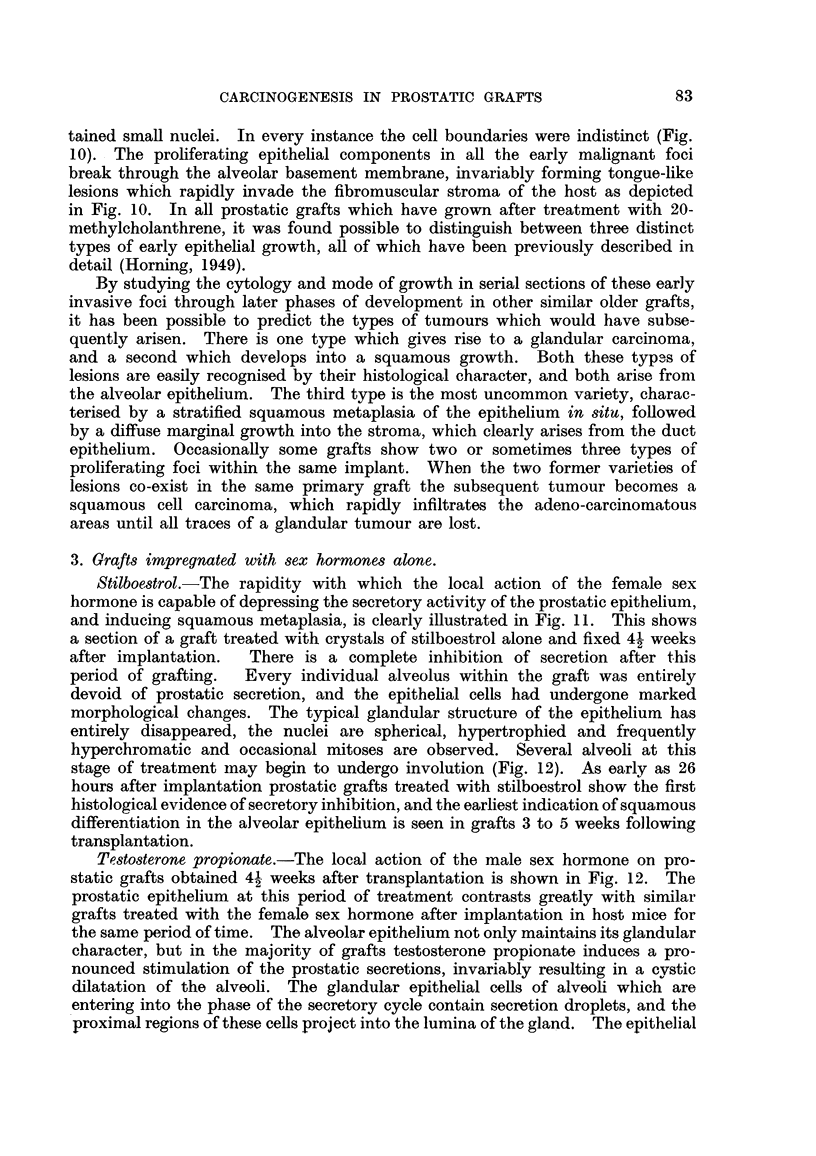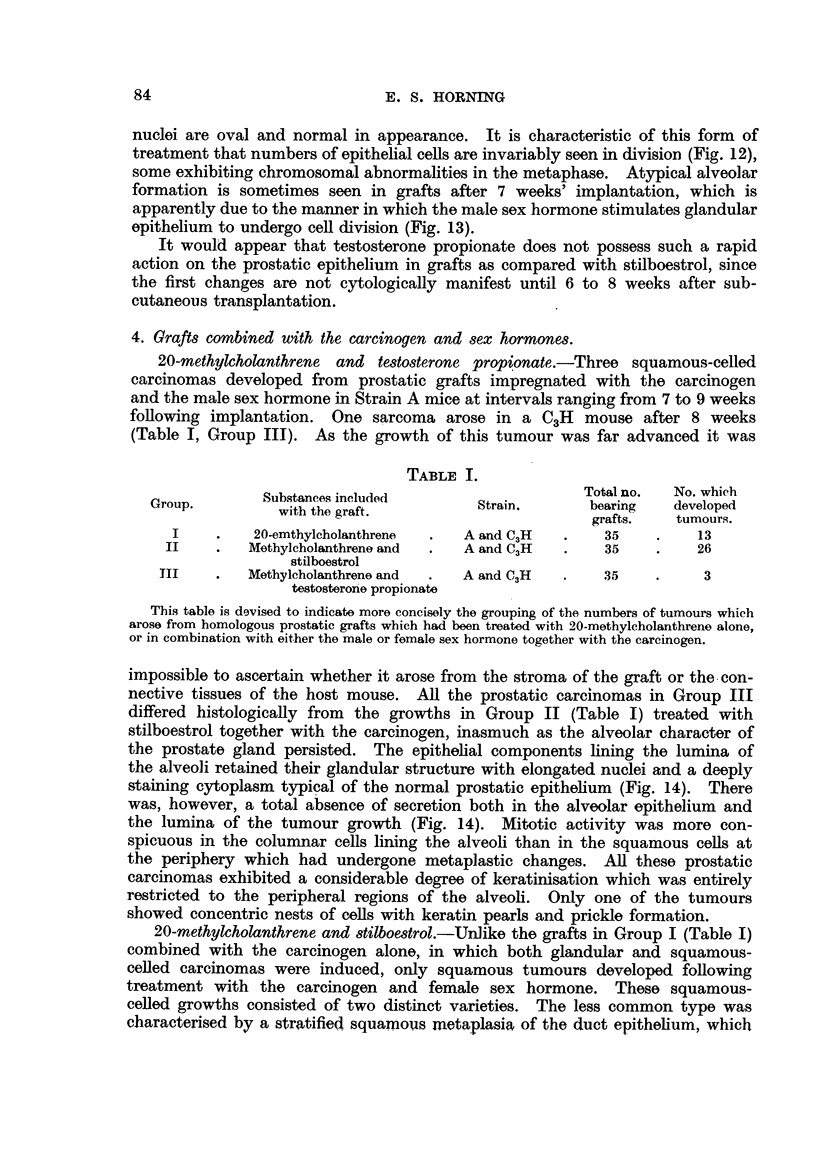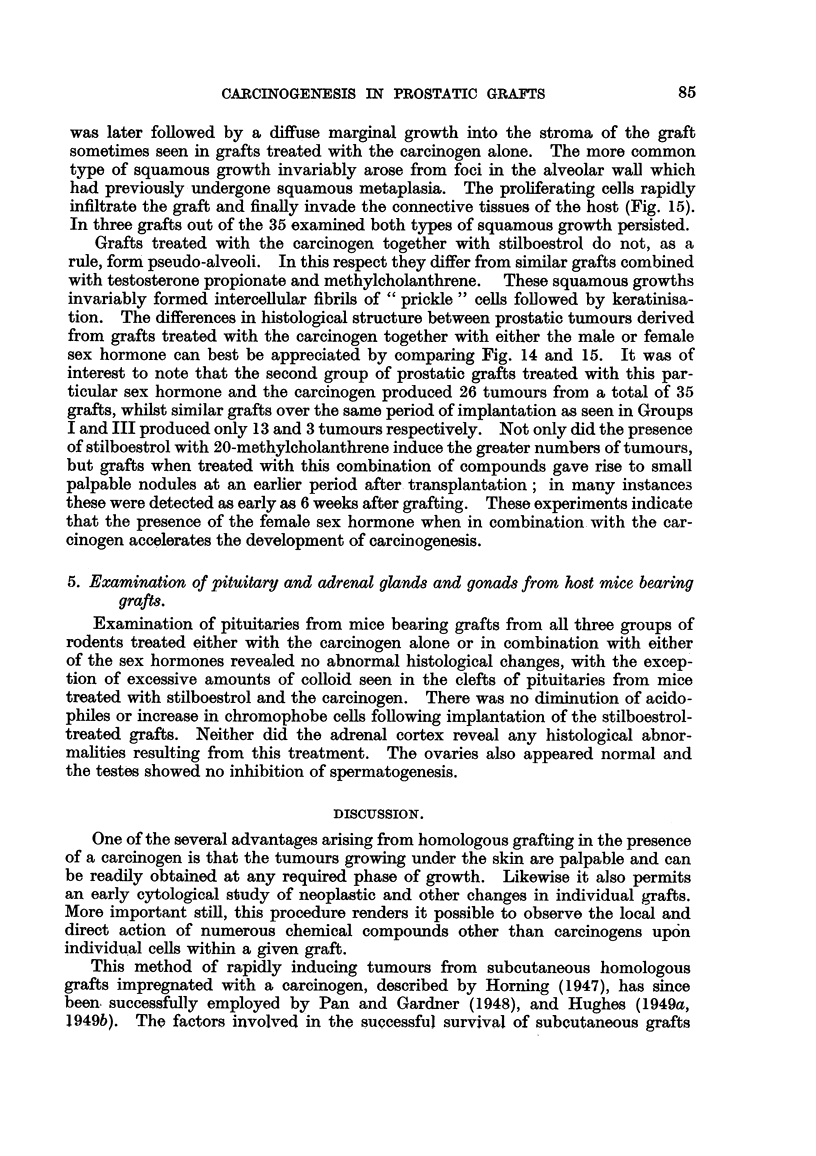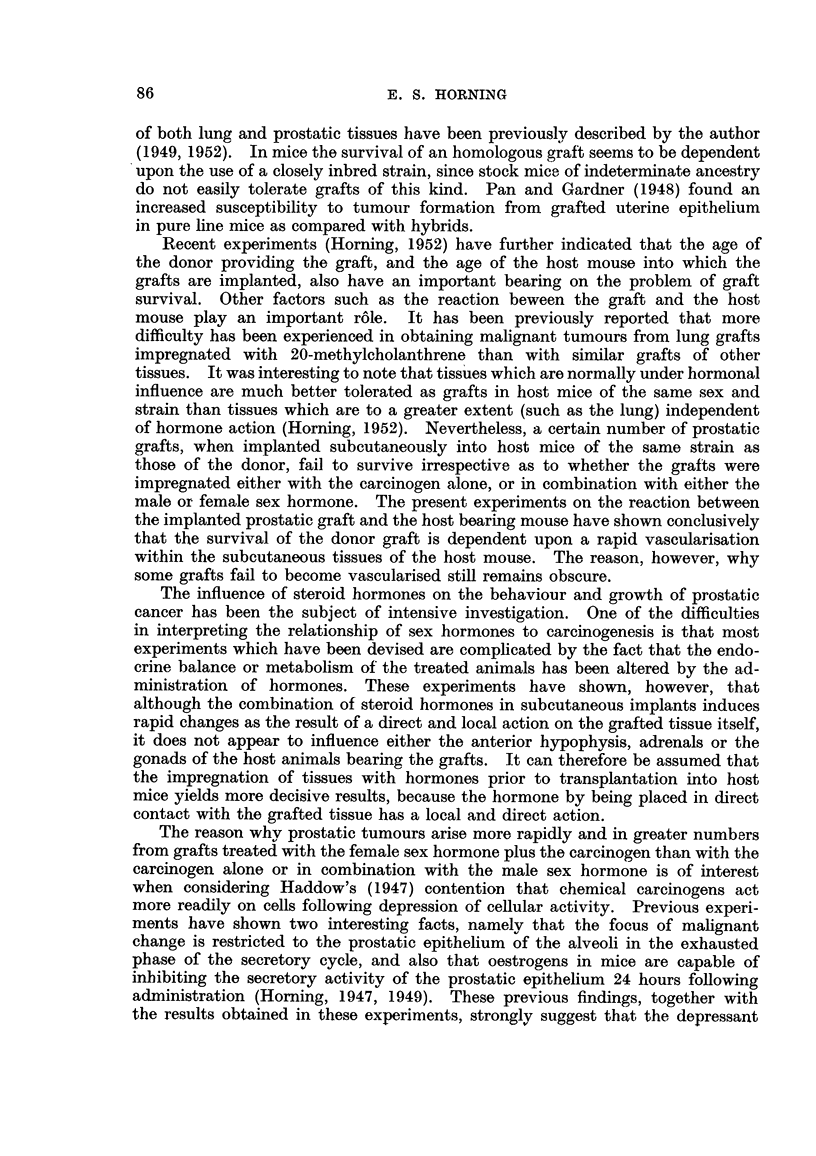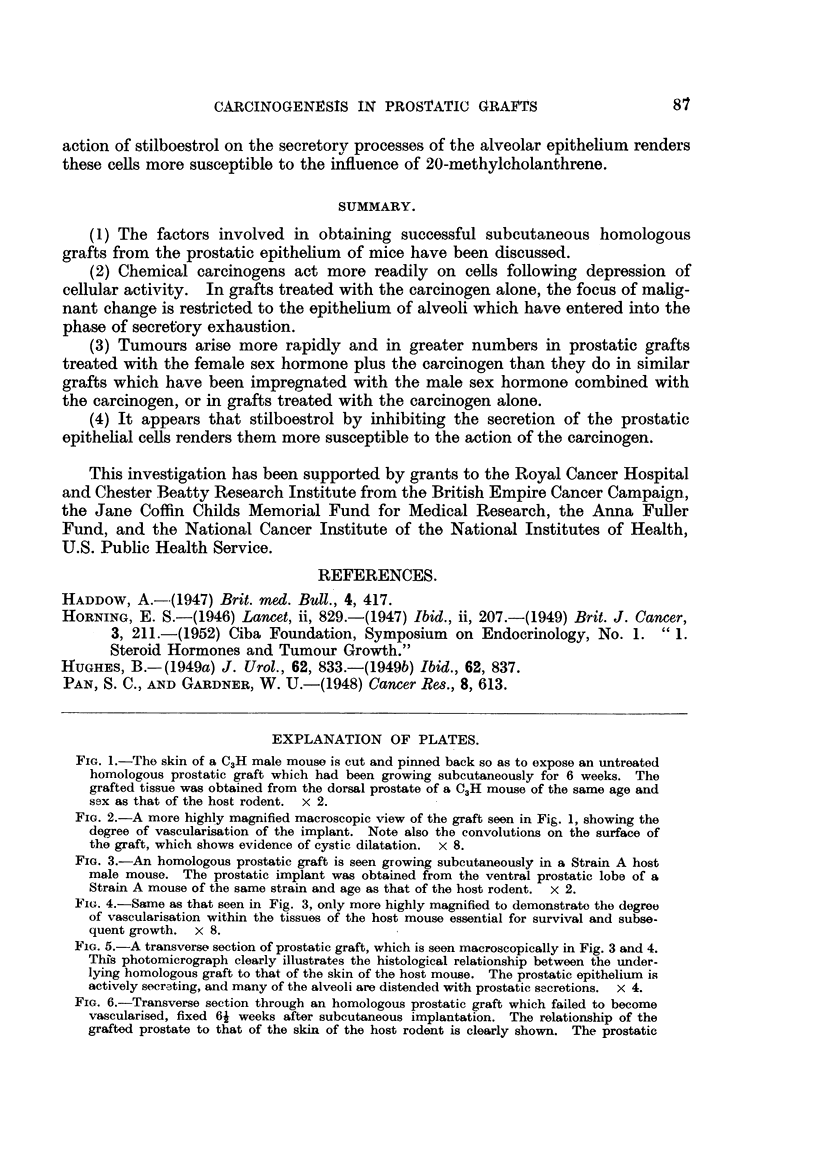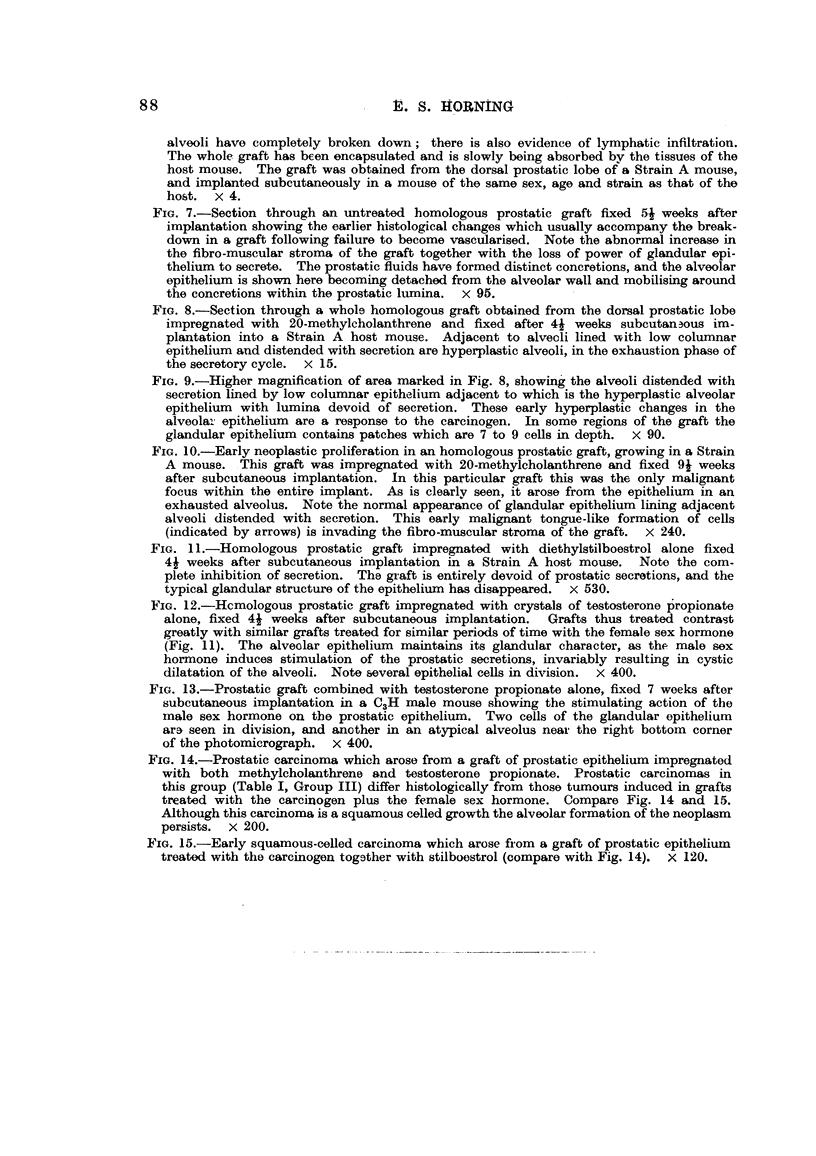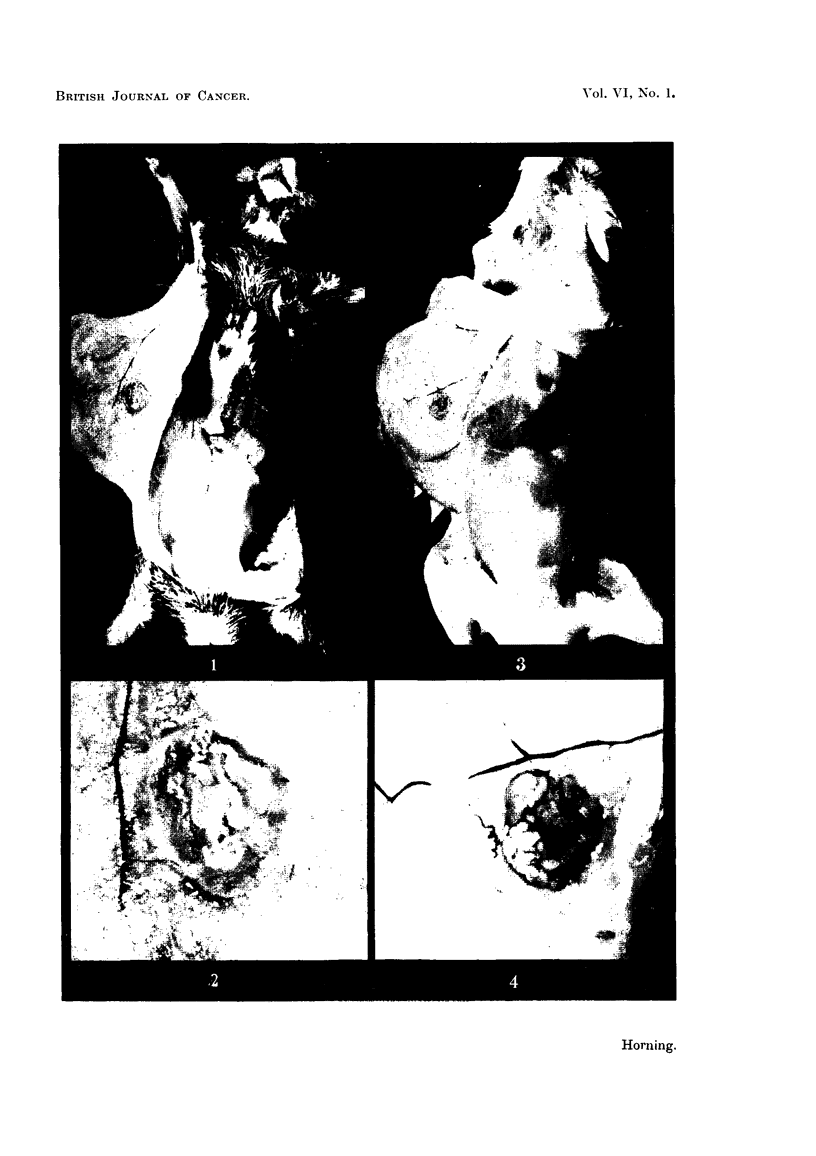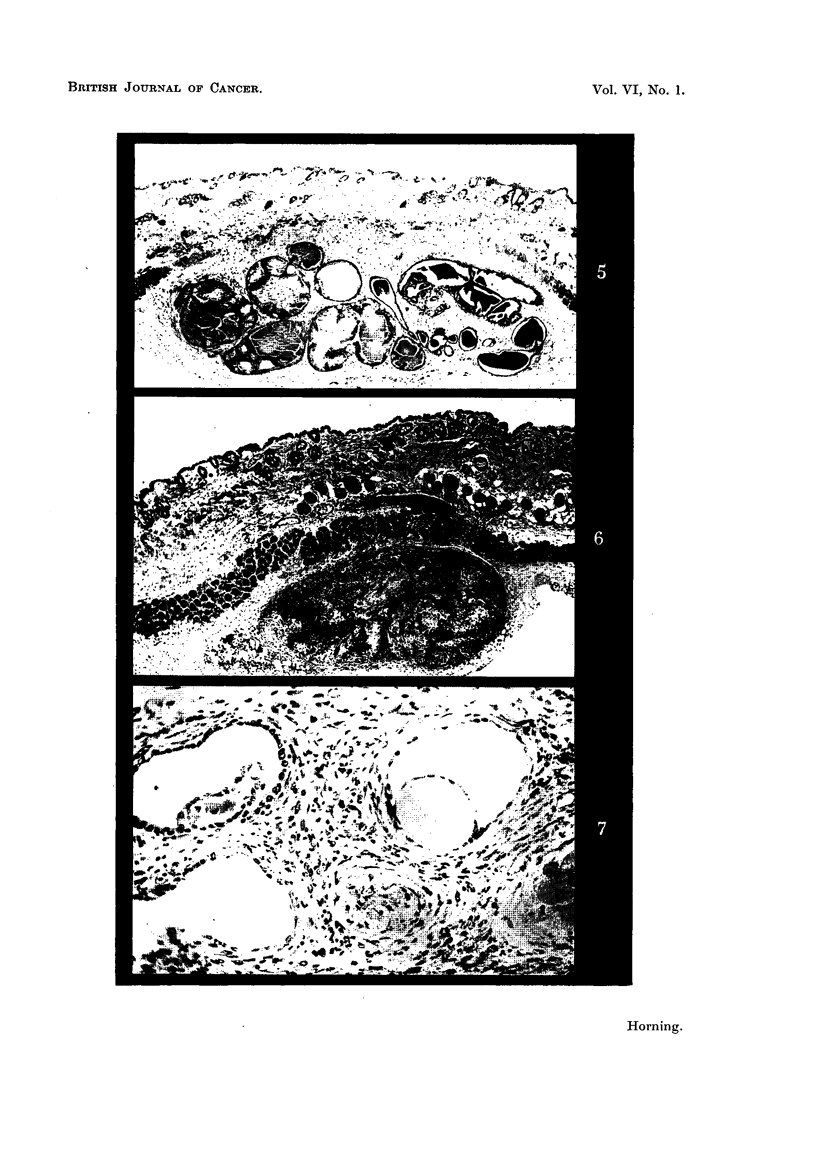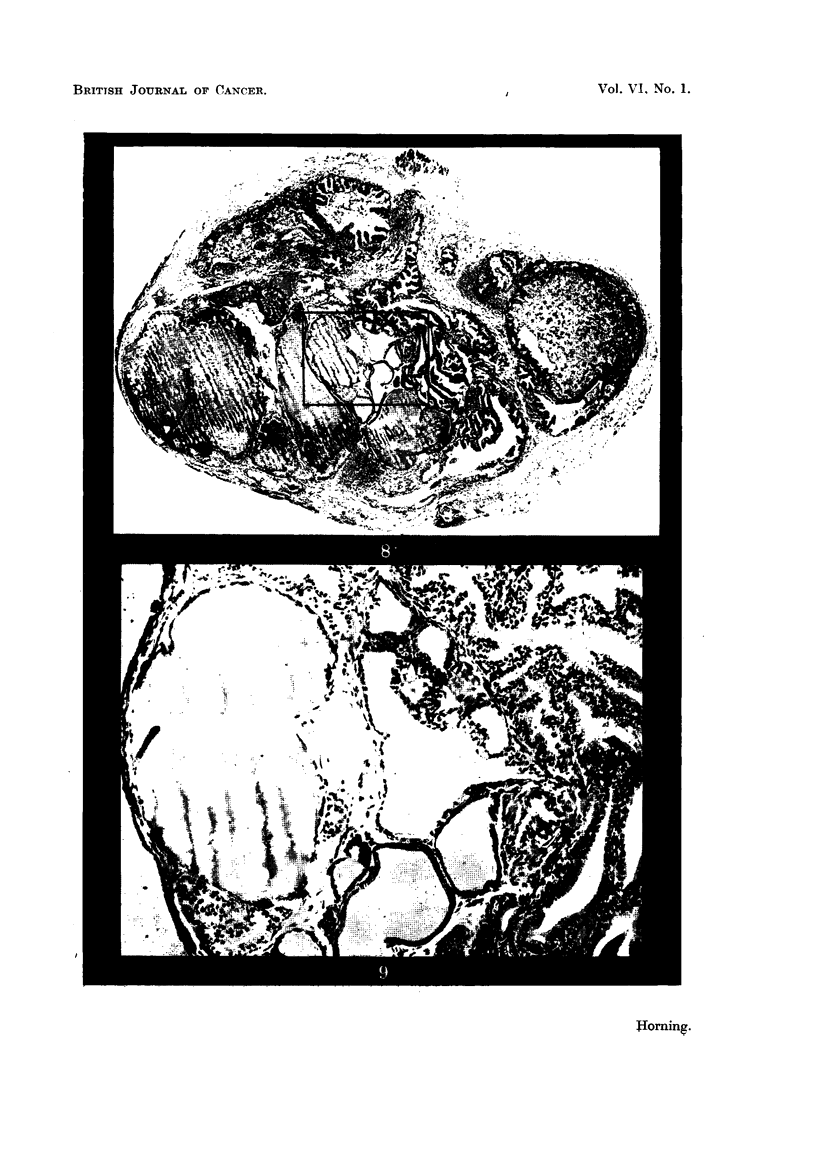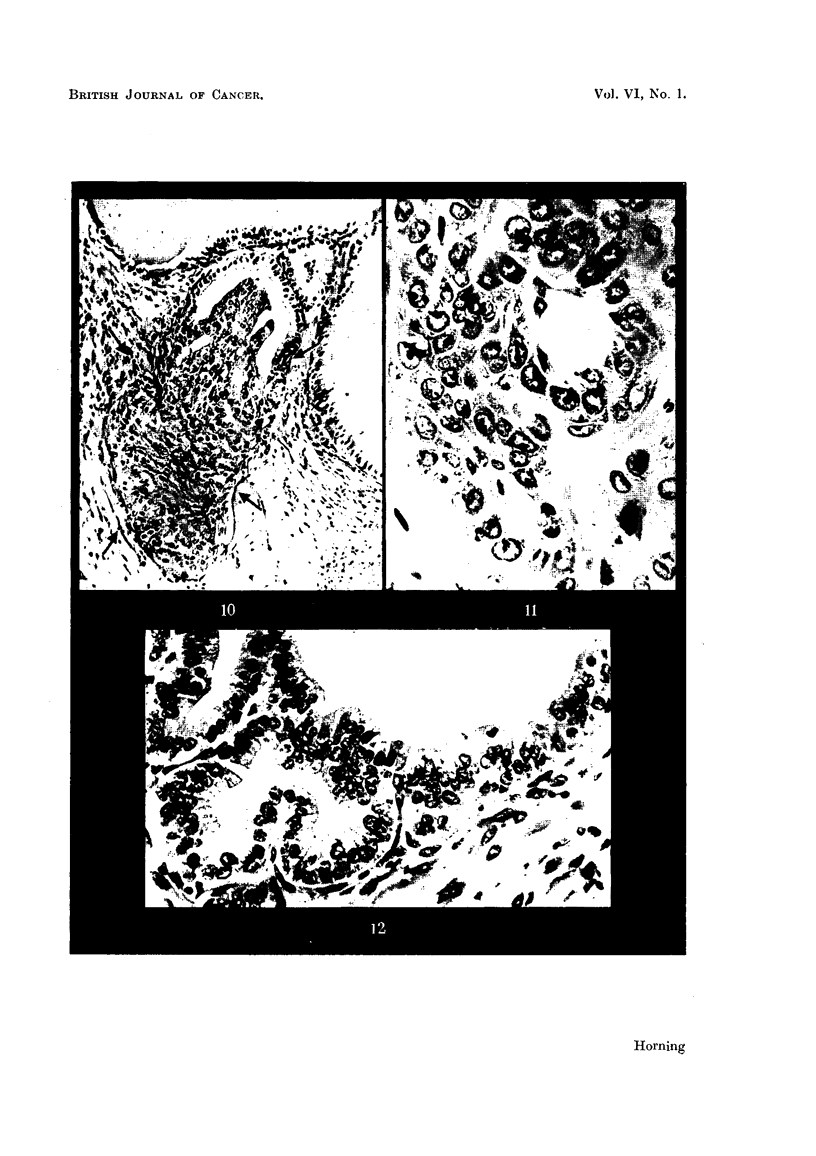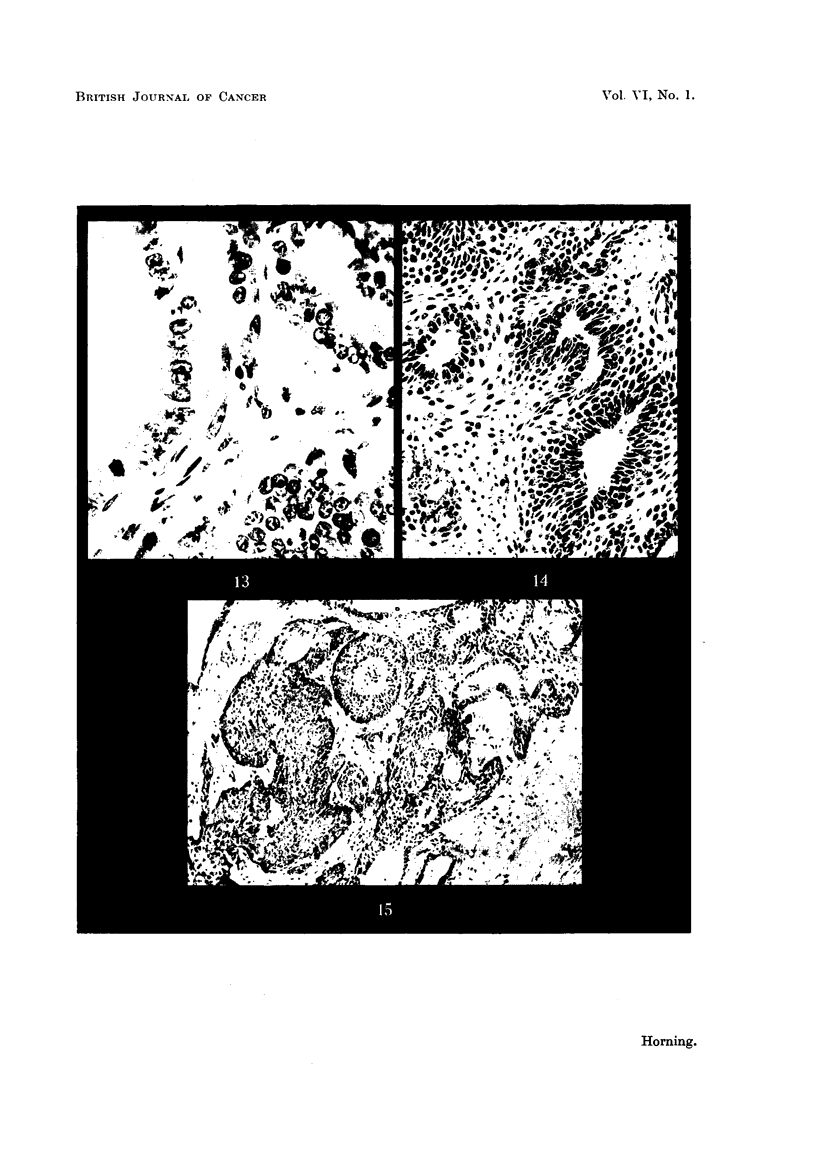# The Local Action of 20-Methylcholanthrene and Sex Hormones on Prostatic Grafts

**DOI:** 10.1038/bjc.1952.8

**Published:** 1952-03

**Authors:** E. S. Horning

## Abstract

**Images:**


### THE LOCAL ACTION OF 20-METHYLCHOLANTHRENE AND SEX HORMONES ON PROSTATIC GRAFTS.

E. S. HORNING.

*From the Chester Beatty Research Institute, The Royal Cancer Hospital, London, S.W.3.*

Received for publication February 13, 1952.

PREVIOUS studies on the histogenesis of neoplasms arising from homologous subcutaneous grafts of mouse prostatic epithelium impregnated with a carcinogen have indicated that the actively secreting prostatic alveolus is never the focus of malignant change (Horning, 1949, 1952). These experiments suggest that the non-secreting resting alveolar cells in the exhaustive phase of the secretory cycle are more susceptible to the local action of the carcinogen than those at the height of secretory activity. In view of these results, which are in accord with the contention of Haddow (1947) that chemical carcinogens act more readily in cells following depression of cellular activity, experiments were devised to compare the depressant action of oestrogen in combination with the carcinogen with the contrary action of testosterone which stimulates the secretion of prostatic epithelium.

This investigation was further extended in order to ascertain if the inclusion of either the male or female sex hormone together with the carcinogen either retards or accelerates the development of carcinogenesis. For this purpose a series of prostatic grafts was prepared with the carcinogen alone, and others with either oestrogen or testosterone in combination with the carcinogen. Since the success of this method is dependent upon homologous grafting, additional experiments were also made to elucidate problems relating to the graft-host relationship.

### TECHNIQUE.

The mouse prostatic epithelium is almost identical in its histology with that of the human (Horning, 1949). Hyperplasia of the prostate gland, however, never occurs spontaneously in rodents, as in man, but a similar condition sometimes arises in mice following prolonged treatment with oestrogens, which induces a pronounced metaplasia of the prostatic epithelium in the dorsal and ventral lobes, involving, as in man, urethral obstruction, retention of urine and hydronephrosis (Horning, 1949).

The method of tumour induction consists in isolating small strips of prostatic epithelium from either the dorsal, ventral and anterior lobes of the prostate gland, impregnating them with crystals of the carcinogen or hormone prior to transplanting them subcutaneously into host mice of the same strain, sex and age, from which the donor tissues were obtained. Details of this method of implantation have previously been described (Horning, 1946, 1947, 1949, 1952).

Male mice belonging to the Strong A and $C_3H$ strains, all approximately under 12 weeks of age, were selected for these experiments. In some instances pieces of prostatic epithelium were treated with crystals of 20-methylcholanthrene alone, and in others the carcinogen was applied with either stilboestrol or testosterone propionate. Another series of prostatic grafts was prepared and was impregnated with either the male or female sex hormone respectively. Grafts treated with the carcinogen alone or in combination with either of the sex hormones were fixed for histological examination at intervals from 4 to 16 weeks following subcutaneous implantation into host mice. Prostatic implants treated with the hormones alone were obtained for examination at intervals ranging from 12 hours to 6 weeks after grafting.

All grafts fixed for histological examination were fixed either in Zenker-formol or in alcoholic Bouin, and were subsequently stained with haematoxylin and eosin.

### RESULTS.

1. *Graft-host relationship.*

The factors involved in the successful survival of homologous grafts growing subcutaneously in host mice will be briefly discussed. It is advantageous that the grafted donor tissue for implantation should be selected from mice of the same strain and age as those of the host animals, and when grafting prostatic epithelium it is particularly beneficial to use male host mice.

Previously it has been shown that grafts transplanted into host mice of indeterminate ancestry have less chance of survival (Horning, 1947, 1952; Pan and Gardner, 1948). In nearly every series of experiments, however, a variable number of prostatic grafts implanted subcutaneously into host mice of the same strain, sex and age as those of the donor fail to survive, and gradually become absorbed by the tissues of the host. Experiments into this aspect of the problem have demonstrated that successful survival of the donor implant is dependent upon a rapid vascularisation of the graft within the tissues of the host mouse, irrespective as to whether the grafts were impregnated with either a carcinogen or sex hormones, or with a combination of both.

Fig. 1 to 4 illustrate the macroscopical appearance of successfully vascularised prostatic grafts growing subcutaneously in both Strain A and $C_3H$ male host mice fixed 6 weeks after implantation. Fig. 2 and 4 are a higher magnification of the same grafts, both of which clearly demonstrate the degree of vascularisation within the tissues of the host mice essential for survival and subsequent growth. A transverse section through one of these grafts (Fig. 4) is shown in Fig. 5, and illustrates the histological relationship between the underlying prostatic graft and the skin of the host-bearing mouse. The prostatic epithelium is healthy and is actively secreting; some of the alveoli are seen distended with prostatic secretion to the point of cystic dilatation. The alveolar epithelium in this particular graft actually shows the various stages in the secretory cycle of the normal prostatic gland.

It is interesting to compare the histology of these successfully vascularised grafts seen in Fig. 1 to 5 with a similar prostatic graft after the same period of implantation, in a strain A mouse, which failed to become vascularised within the host tissues (Fig. 6). The alveoli have completely broken down, there is evidence of a marked lymphocytic infiltration around the periphery of the whole graft, which has become encapsulated, and is in the process of being absorbed by the tissues of the host mouse.

It is relatively easy to detect by palpation non-vascularised grafts, as they are smaller and invariably softer when compared with successfully vascularised prostatic grafts, which are firmer on palpation, larger and invariably cystic in the earlier phases of growth, owing to active secretion of the glandular epithelium.

Fig. 7 is a section through an untreated non-vascularised graft fixed $5\frac{1}{2}$ weeks after implantation, and shows the earlier histological changes which usually accompany the breakdown of the graft. The first histological change seen in grafts after failure to become vascularised is the complete loss of power of the prostatic epithelium to secrete (Fig. 7), also the prostatic fluids, instead of being evenly dispersed throughout the lumina, form distinct concretions similar to those often seen in the human gland. This contrasts with the prostatic secretion seen in successfully vascularised grafts, and also in the normal mouse prostate gland *in situ*, which never forms concretions.

In these early stages of absorption of non-vascularised grafts, the alveolar epithelial cells invariably become detached from the alveolar wall and surround the concretions in a characteristic manner (Fig. 7). Subsequently the alveoli undergo involution, and later the epithelium surrounding the concretions slowly undergoes cellular degeneration and disappears. The prostatic concretions become absorbed by the connective tissues of the host, and this process is accompanied by a pronounced lymphocytic infiltration with final encapsulation of the whole graft as illustrated in Fig. 6.

2. *Grafts implanted with carcinogen alone.*

Because the carcinogen in subcutaneous grafts is placed in direct contact with the living tissue without having to use a solvent, it induces very little foreign body reaction or necrosis. Thus it is possible to study serial sections of the primary grafts, and to trace each invading group of malignant cells back to the individual alveolus from which it had arisen. Histological examination of early neoplasia in some 150 prostatic grafts impregnated with 20-methylcholanthrene alone in previous experiments showed that in no single instance had the actively secreting prostatic epithelium been the focus of malignant change. This process is best appreciated by examining a section through a whole graft of the dorsal prostatic lobe of a Strain A mouse fixed $4\frac{1}{2}$ weeks after implantation, in which adjacent to alveoli lined with a low columnar epithelium and distended with secretions are hyperplastic alveoli, in the exhaustion phase of the secretory cycle (Fig. 8 and 9). The tall columnar epithelial cells, which are characteristic of the exhausted alveoli, are folded and relatively free of secretion probably owing to its absorption by the host tissues, and it is in these alveoli following a phase of mitosis, abnormal cell division and pycnosis that hyperplastic epithelial changes are first detected. These hyperplastic changes in the mouse prostate gland are also accompanied by a pronounced increase in the fibromuscular stroma.

An early neoplastic proliferation in a graft of dorsal prostatic epithelium is seen in Fig. 10. This type of lesion is generally confined to a restricted area in the glandular epithelium of an exhausted alveolus. In this particular implant this was the only malignant focus within the entire graft.

Alveoli distended with secretion and with normal epithelium are seen adjacent to this exhausted alveolus from which this early malignant lesion arose. The individual cells comprising this lesion possessed all the characteristics of malignancy. Some cells were binucleated, others showed abnormal mitosis or contained small nuclei. In every instance the cell boundaries were indistinct (Fig. 10). The proliferating epithelial components in all the early malignant foci break through the alveolar basement membrane, invariably forming tongue-like lesions which rapidly invade the fibromuscular stroma of the host as depicted in Fig. 10. In all prostatic grafts which have grown after treatment with 20-methylcholanthrene, it was found possible to distinguish between three distinct types of early epithelial growth, all of which have been previously described in detail (Horning, 1949).

By studying the cytology and mode of growth in serial sections of these early invasive foci through later phases of development in other similar older grafts, it has been possible to predict the types of tumours which would have subsequently arisen. There is one type which gives rise to a glandular carcinoma, and a second which develops into a squamous growth. Both these types of lesions are easily recognised by their histological character, and both arise from the alveolar epithelium. The third type is the most uncommon variety, characterised by a stratified squamous metaplasia of the epithelium *in situ*, followed by a diffuse marginal growth into the stroma, which clearly arises from the duct epithelium. Occasionally some grafts show two or sometimes three types of proliferating foci within the same implant. When the two former varieties of lesions co-exist in the same primary graft the subsequent tumour becomes a squamous cell carcinoma, which rapidly infiltrates the adeno-carcinomatous areas until all traces of a glandular tumour are lost.

3. *Grafts impregnated with sex hormones alone.*

*Stilboestrol.*—The rapidity with which the local action of the female sex hormone is capable of depressing the secretory activity of the prostatic epithelium, and inducing squamous metaplasia, is clearly illustrated in Fig. 11. This shows a section of a graft treated with crystals of stilboestrol alone and fixed 4½ weeks after implantation. There is a complete inhibition of secretion after this period of grafting. Every individual alveolus within the graft was entirely devoid of prostatic secretion, and the epithelial cells had undergone marked morphological changes. The typical glandular structure of the epithelium has entirely disappeared, the nuclei are spherical, hypertrophied and frequently hyperchromatic and occasional mitoses are observed. Several alveoli at this stage of treatment may begin to undergo involution (Fig. 12). As early as 26 hours after implantation prostatic grafts treated with stilboestrol show the first histological evidence of secretory inhibition, and the earliest indication of squamous differentiation in the alveolar epithelium is seen in grafts 3 to 5 weeks following transplantation.

*Testosterone propionate.*—The local action of the male sex hormone on prostatic grafts obtained 4½ weeks after transplantation is shown in Fig. 12. The prostatic epithelium at this period of treatment contrasts greatly with similar grafts treated with the female sex hormone after implantation in host mice for the same period of time. The alveolar epithelium not only maintains its glandular character, but in the majority of grafts testosterone propionate induces a pronounced stimulation of the prostatic secretions, invariably resulting in a cystic dilatation of the alveoli. The glandular epithelial cells of alveoli which are entering into the phase of the secretory cycle contain secretion droplets, and the proximal regions of these cells project into the lumina of the gland. The epithelial nuclei are oval and normal in appearance. It is characteristic of this form of treatment that numbers of epithelial cells are invariably seen in division (Fig. 12), some exhibiting chromosomal abnormalities in the metaphase. Atypical alveolar formation is sometimes seen in grafts after 7 weeks' implantation, which is apparently due to the manner in which the male sex hormone stimulates glandular epithelium to undergo cell division (Fig. 13).

It would appear that testosterone propionate does not possess such a rapid action on the prostatic epithelium in grafts as compared with stilboestrol, since the first changes are not cytologically manifest until 6 to 8 weeks after subcutaneous transplantation.

4. *Grafts combined with the carcinogen and sex hormones.*

20-*methylcholanthrene and testosterone propionate*.—Three squamous-celled carcinomas developed from prostatic grafts impregnated with the carcinogen and the male sex hormone in Strain A mice at intervals ranging from 7 to 9 weeks following implantation. One sarcoma arose in a $C_3H$ mouse after 8 weeks (Table I, Group III). As the growth of this tumour was far advanced it was

TABLE I.

| Group. | Substances included with the graft. | Strain. | Total no. bearing grafts. | No. which developed tumours. |
|---|---|---|---|---|
| I | 20-emthylcholanthrene | A and $C_3H$ | 35 | 13 |
| II | Methylcholanthrene and stilboestrol | A and $C_3H$ | 35 | 26 |
| III | Methylcholanthrene and testosterone propionate | A and $C_3H$ | 35 | 3 |

This table is devised to indicate more concisely the grouping of the numbers of tumours which arose from homologous prostatic grafts which had been treated with 20-methylcholanthrene alone, or in combination with either the male or female sex hormone together with the carcinogen.

impossible to ascertain whether it arose from the stroma of the graft or the connective tissues of the host mouse. All the prostatic carcinomas in Group III differed histologically from the growths in Group II (Table I) treated with stilboestrol together with the carcinogen, inasmuch as the alveolar character of the prostate gland persisted. The epithelial components lining the lumina of the alveoli retained their glandular structure with elongated nuclei and a deeply staining cytoplasm typical of the normal prostatic epithelium (Fig. 14). There was, however, a total absence of secretion both in the alveolar epithelium and the lumina of the tumour growth (Fig. 14). Mitotic activity was more conspicuous in the columnar cells lining the alveoli than in the squamous cells at the periphery which had undergone metaplastic changes. All these prostatic carcinomas exhibited a considerable degree of keratinisation which was entirely restricted to the peripheral regions of the alveoli. Only one of the tumours showed concentric nests of cells with keratin pearls and prickle formation.

20-*methylcholanthrene and stilboestrol*.—Unlike the grafts in Group I (Table I) combined with the carcinogen alone, in which both glandular and squamous-celled carcinomas were induced, only squamous tumours developed following treatment with the carcinogen and female sex hormone. These squamous-celled growths consisted of two distinct varieties. The less common type was characterised by a stratified squamous metaplasia of the duct epithelium, which was later followed by a diffuse marginal growth into the stroma of the graft sometimes seen in grafts treated with the carcinogen alone. The more common type of squamous growth invariably arose from foci in the alveolar wall which had previously undergone squamous metaplasia. The proliferating cells rapidly infiltrate the graft and finally invade the connective tissues of the host (Fig. 15). In three grafts out of the 35 examined both types of squamous growth persisted.

Grafts treated with the carcinogen together with stilboestrol do not, as a rule, form pseudo-alveoli. In this respect they differ from similar grafts combined with testosterone propionate and methylcholanthrene. These squamous growths invariably formed intercellular fibrils of " prickle " cells followed by keratinisation. The differences in histological structure between prostatic tumours derived from grafts treated with the carcinogen together with either the male or female sex hormone can best be appreciated by comparing Fig. 14 and 15. It was of interest to note that the second group of prostatic grafts treated with this particular sex hormone and the carcinogen produced 26 tumours from a total of 35 grafts, whilst similar grafts over the same period of implantation as seen in Groups I and III produced only 13 and 3 tumours respectively. Not only did the presence of stilboestrol with 20-methylcholanthrene induce the greater numbers of tumours, but grafts when treated with this combination of compounds gave rise to small palpable nodules at an earlier period after transplantation ; in many instances these were detected as early as 6 weeks after grafting. These experiments indicate that the presence of the female sex hormone when in combination with the carcinogen accelerates the development of carcinogenesis.

5. *Examination of pituitary and adrenal glands and gonads from host mice bearing grafts.*

Examination of pituitaries from mice bearing grafts from all three groups of rodents treated either with the carcinogen alone or in combination with either of the sex hormones revealed no abnormal histological changes, with the exception of excessive amounts of colloid seen in the clefts of pituitaries from mice treated with stilboestrol and the carcinogen. There was no diminution of acidophiles or increase in chromophobe cells following implantation of the stilboestrol-treated grafts. Neither did the adrenal cortex reveal any histological abnormalities resulting from this treatment. The ovaries also appeared normal and the testes showed no inhibition of spermatogenesis.

### DISCUSSION.

One of the several advantages arising from homologous grafting in the presence of a carcinogen is that the tumours growing under the skin are palpable and can be readily obtained at any required phase of growth. Likewise it also permits an early cytological study of neoplastic and other changes in individual grafts. More important still, this procedure renders it possible to observe the local and direct action of numerous chemical compounds other than carcinogens upon individual cells within a given graft.

This method of rapidly inducing tumours from subcutaneous homologous grafts impregnated with a carcinogen, described by Horning (1947), has since been successfully employed by Pan and Gardner (1948), and Hughes (1949*a*, 1949*b*). The factors involved in the successful survival of subcutaneous grafts of both lung and prostatic tissues have been previously described by the author (1949, 1952). In mice the survival of an homologous graft seems to be dependent upon the use of a closely inbred strain, since stock mice of indeterminate ancestry do not easily tolerate grafts of this kind. Pan and Gardner (1948) found an increased susceptibility to tumour formation from grafted uterine epithelium in pure line mice as compared with hybrids.

Recent experiments (Horning, 1952) have further indicated that the age of the donor providing the graft, and the age of the host mouse into which the grafts are implanted, also have an important bearing on the problem of graft survival. Other factors such as the reaction beween the graft and the host mouse play an important rôle. It has been previously reported that more difficulty has been experienced in obtaining malignant tumours from lung grafts impregnated with 20-methylcholanthrene than with similar grafts of other tissues. It was interesting to note that tissues which are normally under hormonal influence are much better tolerated as grafts in host mice of the same sex and strain than tissues which are to a greater extent (such as the lung) independent of hormone action (Horning, 1952). Nevertheless, a certain number of prostatic grafts, when implanted subcutaneously into host mice of the same strain as those of the donor, fail to survive irrespective as to whether the grafts were impregnated either with the carcinogen alone, or in combination with either the male or female sex hormone. The present experiments on the reaction between the implanted prostatic graft and the host bearing mouse have shown conclusively that the survival of the donor graft is dependent upon a rapid vascularisation within the subcutaneous tissues of the host mouse. The reason, however, why some grafts fail to become vascularised still remains obscure.

The influence of steroid hormones on the behaviour and growth of prostatic cancer has been the subject of intensive investigation. One of the difficulties in interpreting the relationship of sex hormones to carcinogenesis is that most experiments which have been devised are complicated by the fact that the endocrine balance or metabolism of the treated animals has been altered by the administration of hormones. These experiments have shown, however, that although the combination of steroid hormones in subcutaneous implants induces rapid changes as the result of a direct and local action on the grafted tissue itself, it does not appear to influence either the anterior hypophysis, adrenals or the gonads of the host animals bearing the grafts. It can therefore be assumed that the impregnation of tissues with hormones prior to transplantation into host mice yields more decisive results, because the hormone by being placed in direct contact with the grafted tissue has a local and direct action.

The reason why prostatic tumours arise more rapidly and in greater numbers from grafts treated with the female sex hormone plus the carcinogen than with the carcinogen alone or in combination with the male sex hormone is of interest when considering Haddow's (1947) contention that chemical carcinogens act more readily on cells following depression of cellular activity. Previous experiments have shown two interesting facts, namely that the focus of malignant change is restricted to the prostatic epithelium of the alveoli in the exhausted phase of the secretory cycle, and also that oestrogens in mice are capable of inhibiting the secretory activity of the prostatic epithelium 24 hours following administration (Horning, 1947, 1949). These previous findings, together with the results obtained in these experiments, strongly suggest that the depressant action of stilboestrol on the secretory processes of the alveolar epithelium renders these cells more susceptible to the influence of 20-methylcholanthrene.

### SUMMARY.

(1) The factors involved in obtaining successful subcutaneous homologous grafts from the prostatic epithelium of mice have been discussed.

(2) Chemical carcinogens act more readily on cells following depression of cellular activity. In grafts treated with the carcinogen alone, the focus of malignant change is restricted to the epithelium of alveoli which have entered into the phase of secretory exhaustion.

(3) Tumours arise more rapidly and in greater numbers in prostatic grafts treated with the female sex hormone plus the carcinogen than they do in similar grafts which have been impregnated with the male sex hormone combined with the carcinogen, or in grafts treated with the carcinogen alone.

(4) It appears that stilboestrol by inhibiting the secretion of the prostatic epithelial cells renders them more susceptible to the action of the carcinogen.

This investigation has been supported by grants to the Royal Cancer Hospital and Chester Beatty Research Institute from the British Empire Cancer Campaign, the Jane Coffin Childs Memorial Fund for Medical Research, the Anna Fuller Fund, and the National Cancer Institute of the National Institutes of Health, U.S. Public Health Service.

### REFERENCES.

HADDOW, A.—(1947) *Brit. med. Bull.*, **4**, 417.

HORNING, E. S.—(1946) *Lancet*, ii, 829.—(1947) *Ibid.*, ii, 207.—(1949) *Brit. J. Cancer*, **3**, 211.—(1952) Ciba Foundation, Symposium on Endocrinology, No. 1. " 1. Steroid Hormones and Tumour Growth."

HUGHES, B.—(1949*a*) *J. Urol.*, **62**, 833.—(1949*b*) *Ibid.*, **62**, 837.

PAN, S. C., AND GARDNER, W. U.—(1948) *Cancer Res.*, **8**, 613.

---

### EXPLANATION OF PLATES.

FIG. 1.—The skin of a $C_3H$ male mouse is cut and pinned back so as to expose an untreated homologous prostatic graft which had been growing subcutaneously for 6 weeks. The grafted tissue was obtained from the dorsal prostate of a $C_3H$ mouse of the same age and sex as that of the host rodent. × 2.

FIG. 2.—A more highly magnified macroscopic view of the graft seen in Fig. 1, showing the degree of vascularisation of the implant. Note also the convolutions on the surface of the graft, which shows evidence of cystic dilatation. × 8.

FIG. 3.—An homologous prostatic graft is seen growing subcutaneously in a Strain A host male mouse. The prostatic implant was obtained from the ventral prostatic lobe of a Strain A mouse of the same strain and age as that of the host rodent. × 2.

FIG. 4.—Same as that seen in Fig. 3, only more highly magnified to demonstrate the degree of vascularisation within the tissues of the host mouse essential for survival and subsequent growth. × 8.

FIG. 5.—A transverse section of prostatic graft, which is seen macroscopically in Fig. 3 and 4. This photomicrograph clearly illustrates the histological relationship between the underlying homologous graft to that of the skin of the host mouse. The prostatic epithelium is actively secreting, and many of the alveoli are distended with prostatic secretions. × 4.

FIG. 6.—Transverse section through an homologous prostatic graft which failed to become vascularised, fixed 6½ weeks after subcutaneous implantation. The relationship of the grafted prostate to that of the skin of the host rodent is clearly shown. The prostatic alveoli have completely broken down ; there is also evidence of lymphatic infiltration. The whole graft has been encapsulated and is slowly being absorbed by the tissues of the host mouse. The graft was obtained from the dorsal prostatic lobe of a Strain A mouse, and implanted subcutaneously in a mouse of the same sex, age and strain as that of the host. × 4.

FIG. 7.—Section through an untreated homologous prostatic graft fixed 5½ weeks after implantation showing the earlier histological changes which usually accompany the break-down in a graft following failure to become vascularised. Note the abnormal increase in the fibro-muscular stroma of the graft together with the loss of power of glandular epithelium to secrete. The prostatic fluids have formed distinct concretions, and the alveolar epithelium is shown here becoming detached from the alveolar wall and mobilising around the concretions within the prostatic lumina. × 95.

FIG. 8.—Section through a whole homologous graft obtained from the dorsal prostatic lobe impregnated with 20-methylcholanthrene and fixed after 4½ weeks subcutaneous implantation into a Strain A host mouse. Adjacent to alveoli lined with low columnar epithelium and distended with secretion are hyperplastic alveoli, in the exhaustion phase of the secretory cycle. × 15.

FIG. 9.—Higher magnification of area marked in Fig. 8, showing the alveoli distended with secretion lined by low columnar epithelium adjacent to which is the hyperplastic alveolar epithelium with lumina devoid of secretion. These early hyperplastic changes in the alveolar epithelium are a response to the carcinogen. In some regions of the graft the glandular epithelium contains patches which are 7 to 9 cells in depth. × 90.

FIG. 10.—Early neoplastic proliferation in an homologous prostatic graft, growing in a Strain A mouse. This graft was impregnated with 20-methylcholanthrene and fixed 9½ weeks after subcutaneous implantation. In this particular graft this was the only malignant focus within the entire implant. As is clearly seen, it arose from the epithelium in an exhausted alveolus. Note the normal appearance of glandular epithelium lining adjacent alveoli distended with secretion. This early malignant tongue-like formation of cells (indicated by arrows) is invading the fibro-muscular stroma of the graft. × 240.

FIG. 11.—Homologous prostatic graft impregnated with diethylstilboestrol alone fixed 4½ weeks after subcutaneous implantation in a Strain A host mouse. Note the complete inhibition of secretion. The graft is entirely devoid of prostatic secretions, and the typical glandular structure of the epithelium has disappeared. × 530.

FIG. 12.—Homologous prostatic graft impregnated with crystals of testosterone propionate alone, fixed 4½ weeks after subcutaneous implantation. Grafts thus treated contrast greatly with similar grafts treated for similar periods of time with the female sex hormone (Fig. 11). The alveolar epithelium maintains its glandular character, as the male sex hormone induces stimulation of the prostatic secretions, invariably resulting in cystic dilatation of the alveoli. Note several epithelial cells in division. × 400.

FIG. 13.—Prostatic graft combined with testosterone propionate alone, fixed 7 weeks after subcutaneous implantation in a $C_3H$ male mouse showing the stimulating action of the male sex hormone on the prostatic epithelium. Two cells of the glandular epithelium are seen in division, and another in an atypical alveolus near the right bottom corner of the photomicrograph. × 400.

FIG. 14.—Prostatic carcinoma which arose from a graft of prostatic epithelium impregnated with both methylcholanthrene and testosterone propionate. Prostatic carcinomas in this group (Table I, Group III) differ histologically from those tumours induced in grafts treated with the carcinogen plus the female sex hormone. Compare Fig. 14 and 15. Although this carcinoma is a squamous celled growth the alveolar formation of the neoplasm persists. × 200.

FIG. 15.—Early squamous-celled carcinoma which arose from a graft of prostatic epithelium treated with the carcinogen together with stilboestrol (compare with Fig. 14). × 120.

Horning.